# An autopsy report on multiple system atrophy diagnosed immunohistochemically despite severe ischaemic damage: a new approach for investigation of medical practice associated deaths in Japan

**DOI:** 10.1136/jcp.2009.065060

**Published:** 2009-10-20

**Authors:** M Nakajima, H Kojima, Y Takazawa, N Yahagi, K Harada, K Takahashi, K Unuma, K Yoshida

**Affiliations:** 1 Department of Forensic Medicine, Graduate School of Medicine, The University of Tokyo, Tokyo, Japan; 2 Tokyo Metropolitan Institute for Neuroscience, Tokyo, Japan; 3 Department of Pathology, Graduate School of Medicine, The University of Tokyo, Tokyo, Japan; 4 Department of Emergency Medicine, Graduate School of Medicine, The University of Tokyo, Tokyo, Japan

## Abstract

A 60-year old man with a 10-year history of multiple system atrophy (MSA) was found in respiratory arrest. After 4 months of respiratory support with two episodes of septic shock, he died. Autopsy disclosed severe atrophy of the mesencephalon, brainstem, medulla oblongata and cerebellum. Gallyas–Braak, α-synuclein and ubiquitin-positive inclusions in the cytoplasm of glial cells were evident, despite the severe ischaemic damage due to respiratory arrest and subsequent respiratory support for 4 months. The cause of respiratory arrest was not identified, but could be explained by the natural history of MSA. The bereaved family, who had suspected malpractice, was satisfied with the explanation based on the investigation performed by eight expert doctors, one expert nurse, two coordinator nurses and two lawyers in the model project promoted by the Japanese government.

In 2005, the Ministry of Health, Labor and Welfare of Japan started a model project to investigate medical practice associated death.^1^ In “the model project”, a forensic pathologist, a histopathologist and a clinical expert conduct the autopsy and submit an autopsy report. Additionally, other experts review the medical practices and submit another report. In the local appraisal committee, these doctors, the other independent doctors and lawyers (for the patient’s side and the doctor’s side) discuss the case with reference to the two reports. The cause of death, the review of medical practices and the recommendation for accident prevention are explained to the bereaved family and hospital persons at the same time, and the summary of the report is disclosed.^1^

This report is the first forensic autopsy report on multiple system atrophy (MSA) for investigation of the causes of death using the model project promoted by the Japanese government; additionally we report on the usefulness of using histochemistry for diagnosis of MSA in severely damaged brain tissue. The patient was found in respiratory arrest in a hospital. He died 4 months later with respiratory support. Before the autopsy, we explained to the bereaved family that there was a low possibility of finding the true cause of death because of severe brain damage. However, we found α-synuclein- and Gallyas-positive inclusions in the glia; these are the hallmarks of MSA. Additionally, we review on the mechanism underlying the respiratory arrest of MSA patients; this is a current topic in neurology practice and will be an important forensic topic.

## Case report

A 60-year-old man was diagnosed primarily with spinocerebellar atrophy and later with MSA 10 years ago from gait disturbance. There was no family history of MSA. His younger sister had cared him at home. For 4 years, he had been fed through a gastrostomy tube and breathing was assisted through a tracheostomy. He had hypoglycaemia due to rapid transfer (dumping) of food to the intestine for 1 year, and oesophageal regurgitation for a month. As a result of these difficulties, he was admitted to hospital, but staff could barely communicate with him because of lack of eye contact. The CT image showed severe atrophy of the brainstem, medulla and cerebellum, with fourth ventricular dilatation, consistent with MSA. Forty-seven days after admission, he became febrile, and this was thought to derive from pneumonia resulting from the use of tracheostomy tubing. Four days later, a nurse found him in respiratory arrest and started resuscitation. He regained spontaneous beating under mechanical ventilation, with a flat electroencephalograph.

He underwent two episodes of septic shock before he died 4 months later, with progressive deterioration of respiration, and circulatory failure towards the end. When he died, the sister claimed negligence on the part of the nurse since it had been 2 h since the last airway aspiration when respiratory arrest occurred. Therefore, the hospital recommended her to allow the deceased to undergo the “model project” investigation.

## Autopsy findings

A forensic pathologist, a histopathologist and an emergency doctor performed the autopy on the thin man with height of 165 cm and weight of 50.4 kg at 48 h postmortem. They conferred with the attending physician before the autopsy. The coordinator nurses helped with the autopsy, appraisal processes and the grief services. There was no abnormal external finding, except for a mild bed sore, in the patient who had gastrostomy and tracheostomy tubes. The skeletal muscles appeared atrophic.

The heart (356 g) showed concentric hypertrophy with mild focal fibrosis in left ventricular walls and moderate coronary sclerosis. The right ventricle was moderately infiltrated with a lipid layer. The lungs (469 g and 775 g) demonstrated proportionate oedema but no pneumonia. The thoracic cavities contained 1100 ml and 550 ml of exudates. There was an ulcer scar at the gastric angle.

**Figure 1:**
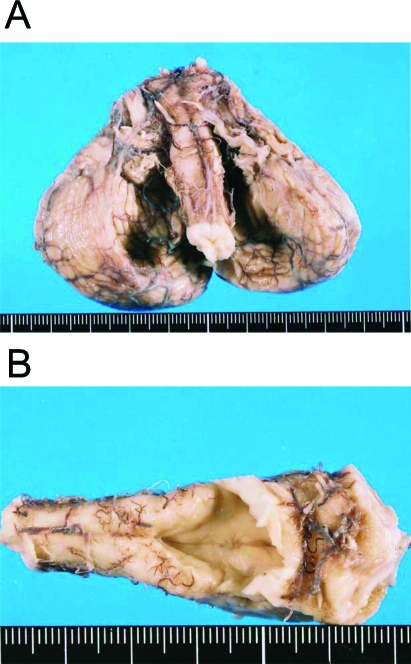
Atrophy in the midbrain, brainstem, medulla and cerebellum from the lower view (A), and dilatation of the 4th ventricle (B).

**Figure 2:**
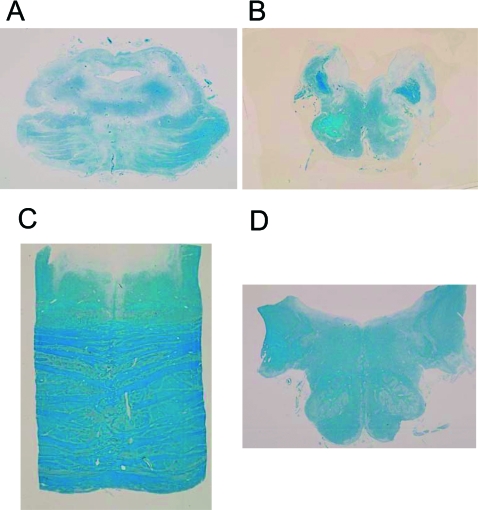
Whole sections of the pons (A) and medulla (B) show severe atrophy and neural loss compared with the pons (C) and medulla (D) of an age-matched control, as shown by the size and reduction in Klüver–Barrera (KB) staining.

**Figure 3:**
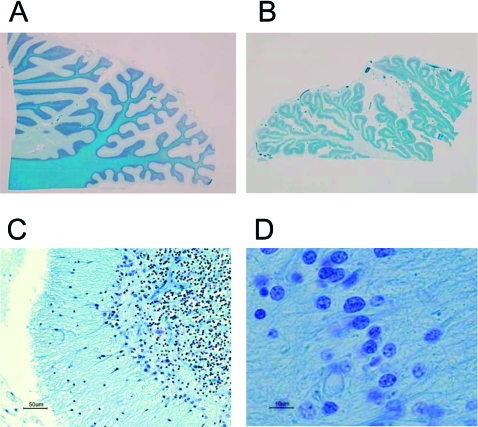
Atrophy found in the cerebellum (A), loss of Purkinje cells (B) and granular cells (C), and infiltration of Bergmann’s glias (D).

The brain (1243 g) showed severe softening and flattening due to diffuse ischaemia. There was severe atrophy in the pons, brainstem, medulla and cerebellum from the lower view (fig 1A), resulting in marked dilatation of the fourth ventricle (fig 1B) and the mesencephalic aquaduct (not shown). The whole sections of the pons and medulla oblongata (fig 2A and B, respectively) showed severe atrophy and neural loss compared with those of an age-matched control (fig 2C and D, respectively), as shown by the size and reduction in Klüver–Barrera (KB) staining. There was severe atrophy of the cerebellum (fig 3A), and loss of Purkinje cells (fig 3B) and granular cells (fig 3C), as well as infiltration of Bergmann’s glias (fig 3D). In the spine, the neuronal loss with the dorsal cord preservation was consistent with sporadic MSA (not shown).

**Figure 4:**
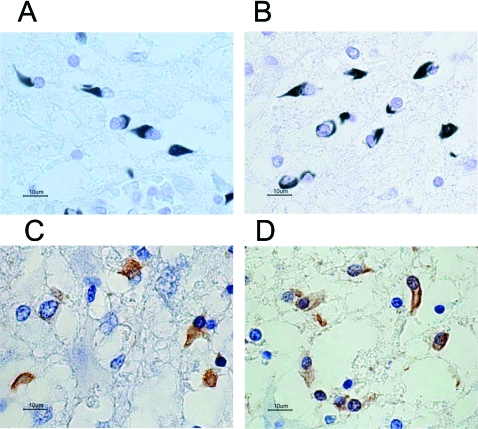
Gallyas–Braak staining shows cytoplasmic inclusions in the cortex (A), and basal ganglia (B). Some inclusions showed positive reactions with anti-α-synuclein (C) and anti-ubiquitin antibody (D).

The histological examination showed severe and diffuse cortex laminar necrosis with fibre gliosis, diffuse neuronal loss and infiltration of macrophages (KB staining). These findings were consistent with ischaemia and reperfusion injury due to respiratory arrest, and resuscitation followed by 4 months of being almost in the state of “respirator brain”. The findings also suggested occurrences of respiratory arrest other than the documented one. Notably, Gallyas–Braak’s staining demonstrated glial cytoplasmic inclusions in the cortex (fig 4A), basal ganglia (fig 4B) and most of the whole brain regions examined (not shown). Some inclusions were positive for anti-α-synuclein (fig 4C) and anti-ubiquitin antibodies (fig 4D). The Gallyas–Braak and α-synuclein-positive inclusions are the hallmark of MSA.^1^ ^8^ ^9^ ^10^ The negative staining for anti-β-amyloid and anti-tau-antibodies (not shown) excluded other α-synucleinopathies such as Alzheimer disease.

Two neurologists submitted a clinical review report to the local appraisal committee, and an expert nurse reported on the review on the nursing care. On the basis of these reports based on clinical review and autopsy, the three autopsy operators, two neurologists, one nurse, two additional doctors and two lawyers discussed and determined the proximal cause of death as MSA, with the direct cause of respiratory arrest as unknown.

## Discussion

MSA is a sporadic neurodegenerative disorder that encompasses olivopontocerebellar atrophy, striatonigral degeneration and Shy–Drager syndrome.^2^ MSA is characterised by cerebellar ataxia, parkinsonism and autonomic dysfunction.^2^ ^3^ ^4^ ^5^ ^6^ ^7^ ^8^ Patients with MSA suffer from ataxia, dysarthria and dysphagia due to degeneration of olivary neurons, pons and spinal cord. The parkinsonisms are manifested as akinesia and muscle rigidity, and autonomic nervous system dysfunction manifests as postural hypotension, vocal cord paralysis and sleep apnoea.^2^ ^3^ ^4^ ^5^ ^6^ ^7^ ^8^

The typical course of MSA in Japanese patients has been reviewed, with an onset of around 55 years, the median times from initial symptoms to combined motor and autonomic dysfunction of 2 years, to a bedridden state of 8 years, and to death of 9 years.^4^ The histopathological hallmark is the formation of α-synuclein-positive and Gallyas-positive argyrophilic glial cytoplasmic inclusions.^2^ ^9^ ^10^ ^11^ ^12^ The inclusions may be also stained with ubiquitin,^9^ as found in this case.

The clinical symptoms and course, marked atrophy of medulla, brainstem, cerebellum and spinal cord, and histological findings, supported the clinical diagnosis of sporadic MSA. The preservation of the Gallyas–Braak and α-synuclein-positive inclusions were unexpected as the brain showed severe and diffuse softening due to respiratory arrest and near “respirator brain” state, and the time lapse before the postmortem (48 h). However, the immunoreactivity was preserved for α-synuclein and ubiquitin, consistent with MSA. Aggregation of α-synuclein underlies α-synucleinopathies including MSA, Parkinson disease, dementia with Lewy bodies (LBs), diffuse LB disease and the LB variant of Alzheimer disease.^9^ ^10^ ^11^ ^12^ ^13^

The direct cause of respiratory arrest was not identified. There was no sign of ischaemia and arrhythmia on ECG or enzyme leakage during the hospitalisation. From the appearance of the mild myocardial lesion, the duration of the respiratory arrest would not have been long. There were two episodes of septic shock, but the focus of inflammation was not identified. The lung oedema at the end-stage may have been caused by the circulatory failure complicated by enhanced vascular permeability and excessive transfusion.

The sister of the patient demanded to know whether asphyxia due to insufficient aspiration of sputa had caused the respiratory arrest. The nurses had not aspirated for 2 h before the respiratory arrest. Moreover, sputa, body temperature, C-reactive protein and leucocyte count had increased over a few days. However, asphyxia was excluded because of the relatively small amount of the sputa aspirated from the airway.

Sleep-disordered breathing in MSA patients is known to cause sudden death. It is hypothesised that hypoventilation may be caused by impaired automatic control of ventilation secondary to degeneration of the pontomedullary respiratory centres; or by stridor and obstructive sleep apnoea due to larynx narrowing secondary to combined vocal cord abductor paralysis and excessive adductor activation during inspiration.^7^

In support of the central mechanism, a histochemical study has confirmed severe loss of putative chemosensitive neurons, accumulation of α-synuclein immunoreactive glial inclusions as well as marked fibre gliosis in the medulla in cases of MSA.^10^ ^11^ ^12^ Additionally, given that autonomic failure is a prominent feature of MSA, a pathological involvement of autonomic neurons would contribute to respiratory arrest in MSA.^3^ ^4^ Rapid eye movement (REM) sleep disorder occurs 90–100% of MSA patients, indicating severe and widespread impairment in the brainstem that regulates REM sleep.^14^ In our case, the atrophy in the brainstem and medulla was severe.

In support of the airway obstruction hypothesis, a clinical study using laryngoscopy under anaesthesia reported vocal cord abductor paralysis (45%) and floppy epiglottis and airway obstruction (55%) of patients with MSA.^8^ It is well known that many patients with MSA suffer from nocturnal laryngeal stridor attributed to paradoxical vocal cord motion (PVCM).^4^ PVCM is the adduction of the vocal cords during inspiration, and it can cause airway obstruction.^4^

It has also been reported that tracheostomy can fatally exacerbate sleep-disordered breathing in MSA, from the observations that the apnoea–hypopnoea index becomes higher after tracheostomy, and all patients with tracheostomies have frequent central sleep apnoeas.^15^ In an epidemiological study, sleep apnoea is an expected event in MSA and the mean time from the onset to death in Japanese patients is 9 years,^5^ whereas the patient in the current case had lived 11 years from the onset of MSA. Collectively, it is reasonable to assume that he died in the natural course of MSA.

From the point of view of the patient’s family, the sister had cared the patient for more than 10 years at home, but she and the attending physician were far away from the hospital when the respiratory arrest happened. Additionally, the sister had criticised the chief nurse on the care given by nurses with less experience than the sister of caring for patients with MSA.

**Take-home messages**

- Multiple system atrophy was diagnosed immunohistochemically despite severe ischaemic brain damage after respiratory arrest followed by 4 months hospitalisation.
- This case was examined by the model project on the investigation of medical practice associated deaths promoted by the Japanese government.

After more than 2 h, she accepted our explanation that there was no negligence on the side of the nurse. She told me that the family would be happy if the findings of this case were to be disclosed in order to prevent the recurrence of similar events. Although this is a case in which the doctors themselves found no evidence of malpractice, but the family of the bereaved suspected that it had occurred, the investigation was able to re-evaluate the case independently.

The model project has been useful in the evaluation as to how we conduct investigations into medical practice associated deaths and explain the findings to the concerned parties. Although the “model project” is useful, it is limited by the intense effort and resources needed to deal with such cases. A characteristic of Japanese culture is to lay the blame on a responsible person, but in view of reluctance of the Japanese to be reviewed by their peers, it will take time before the investigation system can provide safety and confidence in medical practice. Finally, we were able to demonstrate the usefulness of histochemistry in confirming the diagnosis of MSA, despite prolonged survival after hypoxic brain damage.
